# An Outflow Tract Myocardium‐Specific Enhancer at the *Sema3c* Locus During Heart Development

**DOI:** 10.1111/gtc.70130

**Published:** 2026-06-21

**Authors:** Yunce Wang, Yukihiro Harada, Tomoe Ueyama, Daiki Seya, Osamu Nakagawa, Teruhisa Kawamura

**Affiliations:** ^1^ Department of Biomedical Sciences, College of Life Sciences Ritsumeikan University Kusatsu Shiga Japan; ^2^ Research Organization of Science and Technology Ritsumeikan University Kusatsu Shiga Japan; ^3^ Department of Physiology National Defense Medical College Tokorozawa Saitama Japan; ^4^ Departments of Molecular Physiology and Genomic Medicine National Cerebral and Cardiovascular Center Research Institute Suita Osaka Japan

**Keywords:** myocardium, outflow tract, *Sema3c*, transcriptional regulation

## Abstract

*Sema3c* is specifically expressed in the cardiac outflow tract (OFT) of the developing mouse heart and has been implicated in OFT polarization and great artery formation. However, the regulatory basis underlying its spatially restricted expression remains unclear. To investigate the mechanisms underlying OFT‐specific *Sema3c* expression, we utilized chromatin accessibility data from distinct segments of the developing heart and identified a differentially accessible region as an OFT‐specific *Sema3c* enhancer candidate. Unlike previously characterized *Sema3c* enhancers, this region is located distal to the transcription start site. Reporter analysis using transgenic mouse embryos demonstrated that this region exhibits transcriptional activity from E8.5 onward and remains specifically active in the OFT myocardium throughout heart development. We further defined a minimal 603 bp enhancer whose activity depends on GATA binding sites. This enhancer provides insight into the mechanisms underlying spatially restricted *Sema3c* expression involved in OFT development.

## Graphical abstract

Focusing on an OFT‐specific differentially accessible region, in the present study, we defined a distal OFT myocardial enhancer whose activity depends on GATA binding sites. This enhancer provides insight into the mechanisms underlying spatially restricted *Sema3c* expression involved in OFT development.

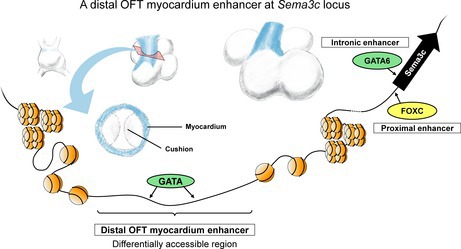

## 1 Introduction

Congenital heart disease (CHD) affects approximately 0.8% of all live births (Mitchell et al. 1971; Reller et al. 2008; Van Der Linde et al. 2011), among which defects involving the cardiac outflow tract (OFT) and the great arteries account for an estimated 30% of cases (Egbe et al. 2014; Thom et al. 2006). During mammalian heart development, the OFT plays a critical role in connecting the developing ventricles to the great arteries and is essential to establish early blood circulation (Neeb et al. 2013; Garry and Olson 2006). The cellular components of the OFT are primarily derived from the anterior second heart field (SHF) and neural crest cells (NCCs) (Kelly et al. 2014; Meilhac and Buckingham 2018). At the four‐chamber stage of heart development, polarization within the OFT and subsequent septation give rise to the separation of the aorta and pulmonary trunk (Hikspoors et al. 2022; Plein et al. 2015). *Sema3c*, a member of the class 3 semaphorin family (Valdembri et al. 2016), is specifically expressed in the OFT during heart development, and loss of *Sema3c* in mice results in abnormal formation of aorta and pulmonary trunk seen in CHDs including persistent truncus arteriosus (PTA) (Feiner et al. 2001). Thus, considerable efforts have been devoted to elucidating the transcriptional regulation of *Sema3c* expression. Loss‐of‐function experiments revealed that *Tbx1 and Nkx2‐5* are involved in OFT‐specific expression of *Sema3c* (Théveniau‐Ruissy et al. 2008; Rana et al. 2014; Yamaguchi et al. 2023). In addition, two enhancers regulating *Sema3c* have been reported so far: an intronic enhancer responsive to GATA6 (Lepore et al. 2006; Kodo et al. 2009) and a proximal enhancer regulated by Foxc1/2 (Kodo et al. 2017). However, mechanisms explaining OFT‐specific *Sema3c* expression remain poorly understood.

We have been conducting a genome‐wide search for enhancers that regulate gene expression in developing hearts. While the details will be presented in another manuscript (Harada et al. in preparation), the present study aims to examine an OFT‐specific differentially accessible region (DAR) located near the *Sema3c* locus as an enhancer candidate. Using *lacZ* reporter transgenic mouse lines, we demonstrated that 1842 bp OFT‐specific DAR exhibits enhancer activity within the OFT myocardium throughout cardiac development. We further defined a minimal 603 bp region that retains OFT‐specific enhancer activity and demonstrated that its activity depends on GATA binding sites but not FOX binding sites. Together, our findings identify an OFT myocardium‐specific enhancer associated with the *Sema3c* locus and provide a clue for investigating the unique transcriptional regulatory programs that underlie OFT development.

## 2 Results

### 2.1 Identification of an OFT‐Specific Accessible Chromatin Region as a Novel *Sema3c* Enhancer Candidate

**FIGURE 1:**
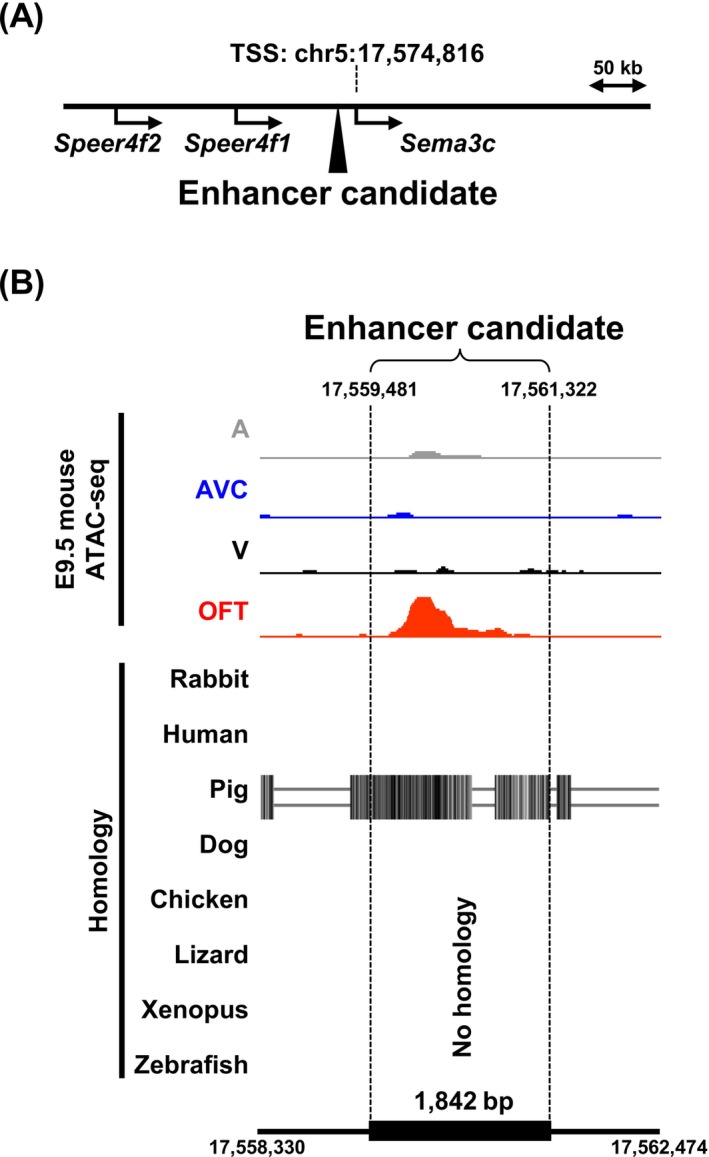
Identification of a *Sema3c* enhancer candidate from an OFT‐specific accessible chromatin region. (A) The closest OFT‐specific DAR upstream of *Sema3c* was identified as an enhancer candidate. The mouse genome assembly version, GRCm38/mm10 was used. (B) ATAC‐seq data of the 1842 bp enhancer candidate located at chr5:17,559,481–17,561,322 are compared among four independent anatomical segments of the E9.5 mouse heart: Atrium (A), atrioventricular canal (AVC), ventricle (V), and outflow tract (OFT). Comparative genomics analysis using the UCSC Genome Browser showed that this candidate exhibits mammalian‐conserved sequence homology.

To investigate how OFT‐specific expression of *Sema3c* is regulated during heart development, we focused on OFT‐specific DAR detected at the looping stage in mice (E9.5). Comparing chromatin accessibility in the genomes of four anatomical segments: OFT, ventricle (V), atrioventricular canal (AVC), and atrium (A), we found the DAR as a *Sema3c* enhancer candidate. This region spans 1842 bp and is located approximately 15 kb upstream of the *Sema3c* transcription start site (Figure 1A). *Sema3c* is the closest gene to this enhancer candidate, and the other neighbor genes have not been reported to be expressed in the developing heart. Comparative genomics analysis using the Vertebrate Multiz Alignment and Conservation tracks of the UCSC Genome Browser revealed that this enhancer candidate is highly conserved among mammals, including humans, but is not conserved in non‐mammalian vertebrates (Figure 1B).

**FIGURE 2:**
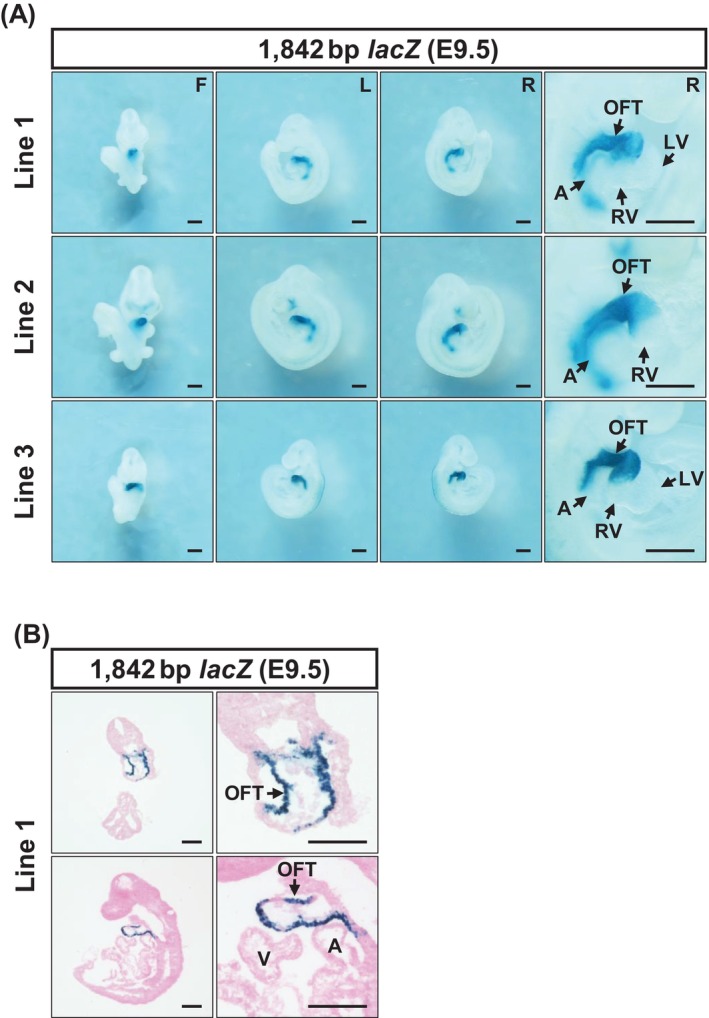
The 1842 bp enhancer candidate exhibits OFT‐specific transcriptional activity in the E9.5 mouse myocardium. (A) Three independent lines of 1842 bp enhancer‐hsp68‐*lacZ* transgenic reporter mouse were analyzed. Representative images of whole mount X‐gal staining (37°C°C for 2 h) are shown. (B) To further assess the cellular localization of reporter activity observed in OFT, cryosections were prepared. *LacZ* activity driven by the 1842 bp DAR was localized to the myocardial layer of the OFT. A, atrium; F, front view; L, left view; LV, left ventricle; OFT, outflow tract; R, right view; RV, right ventricle. Scale bar: 500 μm.

To assess whether this enhancer candidate possesses transcriptional activity, *lacZ* reporter transgenes were generated by pronuclear injection, resulting in three independent transgenic mouse lines (Line 1, Line 2, and Line 3). X‐gal staining performed at 37°C for 2 h on E9.5 embryos from all three lines revealed reproducible reporter activity specifically in the OFT of looping‐stage hearts (Figure 2A). Further analysis of cryosections prepared from X‐gal stained E9.5 embryos demonstrated that reporter activity driven by the enhancer candidate was localized to the myocardial layer of the OFT (Figure 2B). Together, these results indicate that the newly identified OFT‐specific DAR represents an enhancer that may contribute to the regulation of *Sema3c* expression in the OFT myocardium during heart development.

### 2.2 The Enhancer Candidate Exhibits Sustained Myocardium‐Specific Activity During OFT Development

**FIGURE 3:**
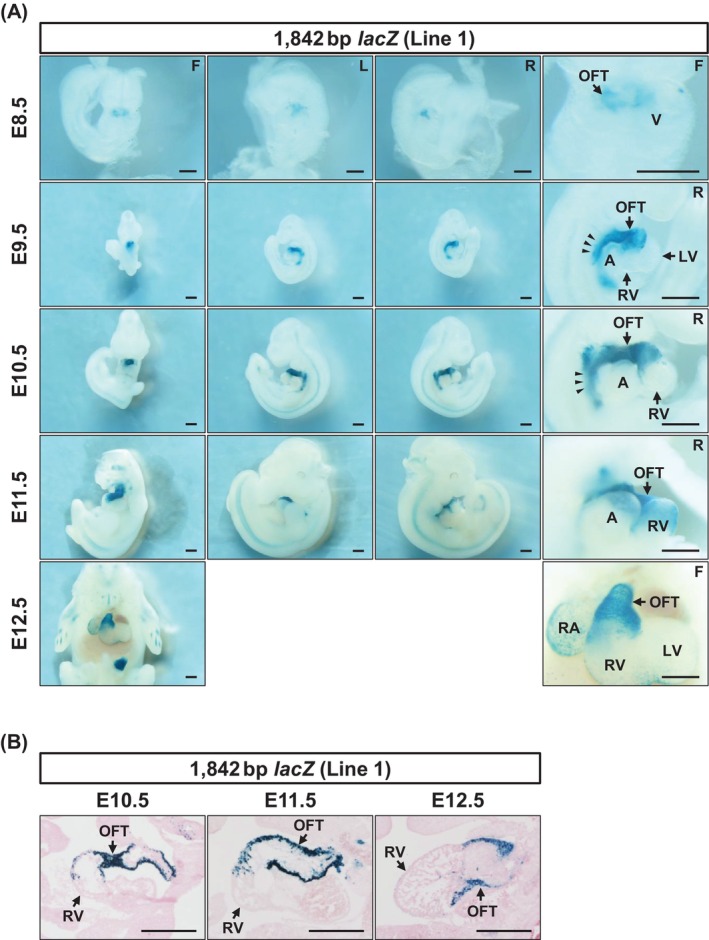
The 1842 bp enhancer exhibits temporally sustained and OFT‐specific transcriptional activity during heart development. (A) Representative images of whole‐mount X‐gal staining of 1842 bp enhancer‐hsp68‐*lacZ* transgenic embryos are shown. At E8.5, reporter activity was detected in the early OFT of the developing heart tube. During the looping stages (E9.5–E11.5), enhancer activity was observed in the OFT but not in the RV. Reporter activity was also detected in a domain corresponding to the SHF at E9.5 and E10.5 (arrowheads). At the four‐chamber stage (E12.5), enhancer activity remained localized to the OFT. Although weak reporter activity was observed in the right atrium in a subset of embryos, analysis of additional independent transgenic lines confirmed that enhancer activity was not reproducibly detected in the right atrium. The E9.5 image is the same as shown in Figure 2A. X‐gal staining was performed at 37°C°C for 2 h (E9.5) or 24 h (E8.5 and E10.5–E12.5). (B) Cryosections prepared at E10.5–E11.5 exhibited that reporter activity was localized to the myocardial layer of the OFT. A, atrium; F, front view; L, left view; LV, left ventricle; OFT, outflow tract; R, right view; RA, right atrium; RV, right ventricle; V, ventricle. Scale bar: 500 μm.

To characterize the temporal dynamics of enhancer activity across different stages of OFT development, *lacZ* reporter activity was examined from E7.5 to E12.5 at 24 h intervals, using one of the transgenic mouse lines (Line1). No reporter activity was detected in embryos at E7.5 stage (Figure S1). At the early stage of OFT formation (E8.5), X‐gal staining performed at 37°C for 24 h revealed reporter activity in a segment corresponding to the early OFT (Figure 3A). Compared with later stages, reporter activity at this stage appeared relatively weak, and no reporter activity was detected in other cardiac segments nor outside the heart at this stage (Figure 3A).

During the OFT elongation stages (E9.5–E11.5), X‐gal staining (E9.5, 37°C for 2 h; E10.5 and E11.5, 37°C for 24 h) revealed highly consistent and spatially restricted enhancer activity (Figure 3A). At these stages, reporter activity was detected exclusively in the OFT and was absent from the right ventricle, which is also derived from the SHF. Outside the heart, enhancer activity was also observed at E9.5 and E10.5 around a posterior wall region corresponding to the dorsal pericardium (arrowheads), a structure closely associated with OFT and right ventricular development (Figures 2A and 3A) (Stefanovic et al. 2020).

At E12.5, corresponding to the early four‐chamber stage, X‐gal staining (37°C for 24 h) demonstrated that enhancer activity remained restricted to the OFT (Figure 3A). No reproducible signal was observed in the right ventricle, consistent with the spatial pattern observed at earlier looping stages. Although embryos from Line 1 exhibited weak reporter activity in the right atrium at E12.5, analysis of Line 2 confirmed that enhancer activity was not reproducibly detected in the right atrium at this stage (Figure S2).

Cryosection analysis further demonstrated that, from E9.5 to E12.5, enhancer‐driven reporter activity was confined to the myocardial layer of the OFT and did not extend into the right ventricular myocardium (Figures 2B and 3B). Notably, reporter activity was not detected in the OFT cushions, despite endogenous *Sema3c* expression in this region (Figures 2B and 3B). Considering these results, we can conclude that the enhancer candidate serves as the *Sema3c* OFT enhancer exhibiting sustained OFT myocardium‐specific activity.

### 2.3 A 603 bp Minimal Enhancer Retains OFT‐Specific Transcriptional Activity

**FIGURE 4:**
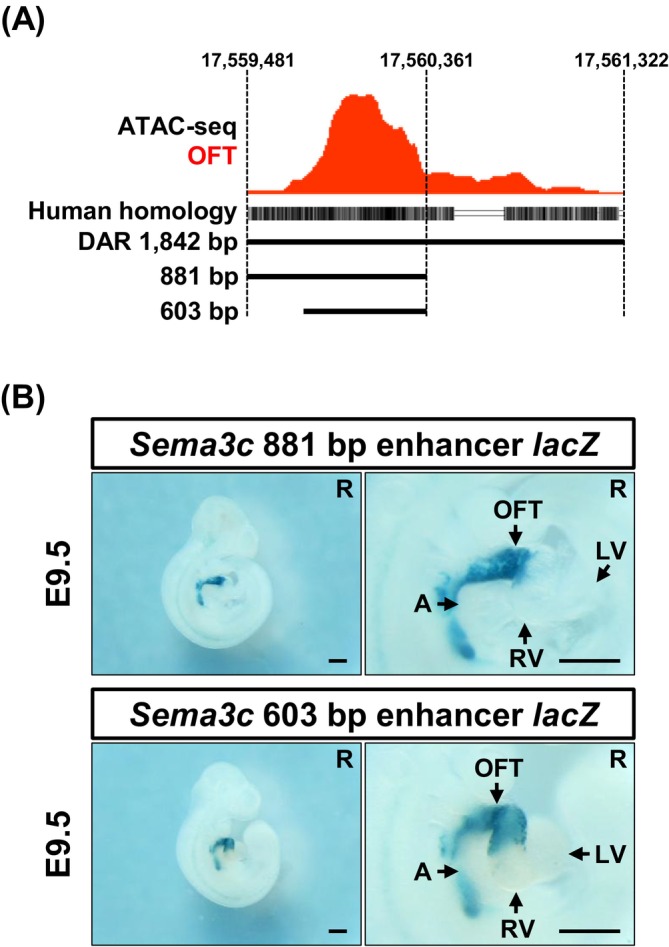
A 603 bp minimal enhancer exhibits OFT‐specific activity. (A) Two truncated enhancer fragments (881 and 603 bp) are derived from the OFT‐specific DAR based on chromatin accessibility profiles. (B) Both minimal enhancer fragments drove OFT‐specific reporter activity in F0 transient transgenic embryos. The spatial pattern of reporter activity driven by the 881 bp (*n* = 2/2) and 603 bp (*n* = 5/8) fragments is shown. A, atrium; LV, left ventricle; OFT, outflow tract; R, right view; RV, right ventricle. Scale bar: 500 μm.

Although the 1842 bp enhancer exhibited clear transcriptional activity, its length is relatively long compared with typical functional enhancers (Blackwood and Kadonaga 1998; Li and Wunderlich 2017), prompting us to investigate whether a shorter fragment could retain enhancer activity. ATAC‐seq peak profile of the 1842 bp enhancer region showed that chromatin accessibility was not uniform across the region, displaying an asymmetry with greater accessibility in its distal half (Figure 4A). To test whether a shorter enhancer fragment could retain activity, we first removed the low‐accessibility region of the 1842 bp enhancer and generated an 881 bp enhancer candidate fragment. F0 reporter analysis at E9.5, with X‐gal staining (37°C for 24 h), confirmed that this 881 bp fragment exhibited OFT‐specific activity comparable to that of the 1842 bp enhancer (Figure 4). The result above supports our hypothesis that enhancer activity correlates with chromatin accessibility.

To define the minimal region required for enhancer activity, we further removed the low‐accessibility portion and generated a 603 bp enhancer candidate fragment (Figure 4A). F0 reporter analysis with X‐gal staining (37°C for 6 h) revealed that this 603 bp fragment exhibited an OFT‐specific *lacZ* expression pattern (E9.5) equivalent to those of the 1842 and 881 bp enhancers (Figure 4). Further deletion attempts failed to produce this pattern comparable to that of the 1842 bp enhancer (Figure S3). These findings indicate that the 603 bp fragment represents the minimal *Sema3c* enhancer capable of driving OFT‐specific transcriptional activity.

### 2.4 GATA Binding Site Dependent Activity of the *Sema3c* 603 bp Enhancer

**FIGURE 5:**
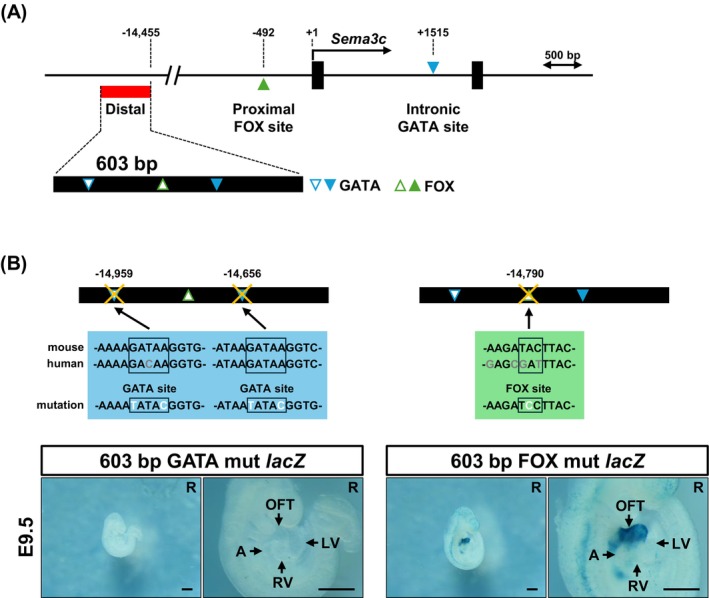
The activity of the *Sema3c* 603 bp enhancer depends on GATA transcription factor binding sites. (A) Genomic location of a novel distal enhancer of *Sema3c*. According to the previous reports, transcription factor binding sites were annotated in the 5′ promoter‐proximal region and within intron 1 of the mouse *Sema3c* gene. The 603 bp enhancer (red box) identified in this study is located approximately 15 kb upstream of the *Sema3c* transcription start site. Motif analysis of the 603 bp enhancer using the JASPAR database (a relative profile score threshold of > 95%) identified enriched transcription factor binding motifs of GATA and FOX families. Open symbols indicate motifs specific to the mouse enhancer sequence, whereas filled symbols denote motifs conserved in humans. (B) The 603 bp enhancer contains two GATA binding sites. Mutation of these sites in F0 embryos resulted in a complete loss of enhancer activity. In contrast, mutation of a single FOX binding site in this enhancer did not affect reporter activity. X‐gal staining was performed at 37°C°C for 24 h (GATA mutation) and 6 h (FOX mutation). A, atrium; LV, left ventricle; OFT, outflow tract; R, right view. Scale bar: 500 μm.

Two cardiac enhancers regulating *Sema3c* expression have been previously characterized: a GATA6‐dependent intronic enhancer (Lepore et al. 2006; Kodo et al. 2009) and a Foxc1/2‐responsive proximal enhancer (Kodo et al. 2017). In contrast to these enhancers, both of which are located close to the transcription start site (TSS), the enhancer identified in this study is located at a distal position upstream of the *Sema3c* locus (Figure 5A). We next performed motif analysis of the distal 603 bp *Sema3c* enhancer. Using the JASPAR 2024 database, we examined predicted binding motifs for FOX and GATA transcription factor families, which have been reported to regulate previously characterized *Sema3c* enhancers. Two GATA and one FOX binding motifs were identified within the 603 bp enhancer (Figure 5B).

Considering previous reports implicating GATA and FOX family transcription factors as upstream regulators of *Sema3c* enhancers (Lepore et al. 2006; Kodo et al. 2009, 2017), we next examined the functional contribution of these motifs by introducing site‐directed mutations into the predicted binding sites within the 603 bp enhancer (Figure 5B). Reporter constructs carrying mutations in either the GATA binding sites or the FOX site were analyzed by F0 transgenic reporter assays. Mutation of the two predicted GATA binding sites resulted in a complete loss of reporter activity in E9.5 embryos following X‐gal staining at 37°C for 24 h. This result indicated that the transcriptional activity of the 603 bp enhancer depends on intact GATA binding sites (Figure 5B). In contrast, mutation of the single predicted FOX binding site did not abolish enhancer activity. In addition, no ectopic *lacZ* expression was observed in F0 reporter embryos carrying each mutant enhancer, compared to the wild‐type enhancer (Figure 5B). Together, these results indicate that GATA binding sites are required for the transcriptional activity of the distal *Sema3c* 603 bp enhancer in the OFT myocardium.

## 3 Discussion

In this study, we validated an OFT‐specific DAR upstream of the *Sema3c* locus (Figure 1). Using transgenic *lacZ* reporter mouse lines, we demonstrated that this region exhibits transcriptional activity in the cardiac OFT, thereby defining an OFT myocardium‐specific enhancer of *Sema3c* (Figures 2 and 3). Through progressive truncation, we further defined a minimal 603 bp enhancer that retains OFT‐specific transcriptional activity (Figures 4 and S3). Mutation of GATA family transcription factor binding sites within this minimal region resulted in a complete loss of enhancer activity, indicating that enhancer function is dependent on GATA transcription factor binding (Figure 5).

Temporal analysis of reporter embryos from E8.5 to E12.5 revealed that enhancer activity is initiated at the onset of OFT formation and subsequently maintained throughout its development, with activity consistently restricted to the OFT myocardium (Figure 3). Although weak X‐gal signals were detected outside the OFT (Figure 3), strong transcriptional activity within the heart was reproducibly confined to the OFT across independent transgenic lines (Figure S2). In future studies, deleting the *Sema3c* OFT enhancer will be important to examine the changes in *Sema3c* expression and determine its potential impact on OFT morphogenesis. Notably, enhancer activity was not detected in the OFT cushions during the E9.5–E12.5 period (Figures 2B and 3B), despite endogenous *Sema3c* expression in this tissue (Plein et al. 2015). This finding suggests that *Sema3c* expression in the OFT myocardium and cushions is governed by distinct transcriptional regulatory mechanisms. Previous studies reported that *Sema3c* expression in neural crest‐derived, Wnt1‐positive OFT cushions contributes to OFT septation (Plein et al. 2015). Meanwhile, *Sema3c* is expressed in the SHF independently of Wnt signaling (Rammah et al. 2022). Analysis of early developmental stages further showed that the 1842 bp *Sema3c* enhancer is not active in the SHF outside the OFT at E8.5, but becomes detectable by E9.5 and persists through E10.5 (Figure 3) (Kelly 2023). Although the anterior SHF contributes to both the OFT and right ventricle (Meilhac and Buckingham 2018; Verzi et al. 2005; Stefanovic et al. 2020), the 1842 bp *Sema3c* enhancer did not exhibit activity in the right ventricle at any stage examined (Figure 3). These observations suggest that cardiac progenitors in the anterior SHF may undergo further regional or lineage diversification during cardiac looping stages. Lineage‐tracing approaches may help to clarify the distinct developmental trajectories underlying OFT and right ventricular formation.

GATA6 and Foxc1/2 have been shown to regulate *Sema3c* transcription through previously characterized enhancer elements located near the transcription start site (Lepore et al. 2006; Kodo et al. 2009, 2017). Our results show that the activity of the distal 603 bp *Sema3c* OFT enhancer depends on GATA transcription factor binding sites (Figure 5). Previous studies have demonstrated that GATA family transcription factors are closely associated with chromatin accessibility, and that loss of GATA factors leads to reduced or abolished accessibility at their target regulatory regions (Heslop et al. 2021; Yamaguchi et al. 2023). It is therefore possible that GATA transcription factors contribute to the establishment or maintenance of OFT‐specific chromatin accessibility at the distal *Sema3c* enhancer. However, the presence of GATA binding sites alone cannot explain the OFT myocardium‐specific activity of the enhancer, as GATA factors are broadly involved in cardiac gene regulation (Charron and Nemer 1999). In addition, weak and discontinuous *lacZ* expression was observed in several enhancer candidates shorter than 603 bp (Figure S3), although GATA transcription factor binding sites were present in these candidates. We therefore speculate that additional, yet unidentified regulatory factors cooperate with GATA transcription factors to confer the spatial specificity of this enhancer. Identifying such factors will be an important direction for future investigation. In contrast to the proximal enhancer reported by Kodo et al. (2017), mutation of the FOX site did not affect the activity of the 603 bp *Sema3c* OFT enhancer (Figure 5B). This may result from the lack of functionality at this FOX site, which is not conserved in humans, unlike the 603 bp enhancer exhibiting high sequence conservation across mammals. Furthermore, since *Foxc1/2* are expressed in the non‐myocardium of the OFT, they are unlikely to regulate this newly identified *Sema3c* OFT enhancer, which is not active in the cushion. Previous studies have reported that *Tbx1* and *Nkx2‐5* are associated with *Sema3c* expression in the OFT (Théveniau‐Ruissy et al. 2008; Rana et al. 2014; Yamaguchi et al. 2023). These transcription factors, along with their downstream effectors, may cooperate with GATA factors to establish the OFT‐myocardium‐specific activity of this enhancer. Interestingly, *Sema3c* expression is controlled by multiple enhancers with distinct regulatory upstream factors during OFT development.

Although the precise transcriptional regulatory mechanisms remain to be fully defined, the distinctive properties of the *Sema3c* OFT enhancer provide a useful tool for dissecting cellular heterogeneity between OFT myocardium and OFT cushions, as well as between OFT myocardium and right ventricular myocardium. In addition, its activity within the SHF may facilitate further refinement of myocardial progenitor classification and lineage tracing in this region. Collectively, the enhancer identified in this study expands the current understanding of transcriptional regulation during OFT development.

## 4 Experimental Procedures

### 4.1 *lacZ* Reporter Analysis

Animal experiments were performed with the approval of the institutional animal care and use committee of Ritsumeikan University (BKC2023‐033). To generate *lacZ* reporter transgenic (*T*_g_) mice, linearized plasmid constructs were injected into B6D2F1/Slc (from Japan SLC Inc.) fertilized eggs.

Enhancer candidate–hsp68 promoter‐nls‐*lacZ* transgenic mice were maintained on a C57BL/6NCrSlc background (Japan SLC Inc.). Embryos were collected at the indicated developmental stages and genotyped as previously described. *lacZ* reporter activity in embryonic hearts was detected by X‐gal staining (Ihara et al. 2020).

For X‐gal staining, embryos at E8.5–E9.5 were pre‐fixed in 4% paraformaldehyde (PFA) in phosphate‐buffered saline (PBS) on ice for 15 min, whereas embryos at E10.5–E12.5 were pre‐fixed in 4% PFA/PBS on ice for 30 min. Following X‐gal staining, all embryos were post‐fixed in 4% PFA/PBS at 4°C for 16 h prior to imaging.

### 4.2 Plasmid Construction

Genomic DNA fragments corresponding to enhancer candidate regions for mouse *Sema3c* were amplified by PCR and cloned into the pT2AL200R175 plasmid containing the hsp68 promoter‐nls‐*lacZ* reporter cassette using the In‐Fusion HD EcoDry Cloning Kit (Takara Bio, 639690).

Primer sequences used for PCR amplification (excluding In‐Fusion adapter sequences) were as follows:

for the 1842 bp enhancer, forward 5′‐GCTGACTGTGGGAGTCTCAA‐3′ and reverse 5′‐TTGCGGGAAAGACAAGGTCA‐3′;

for the 881 bp enhancer, forward 5′‐GCTGACTGTGGGAGTCTCAA‐3′ and reverse 5′‐CATTTAATAAGTTAACAAGA‐3′;

for the 603 bp enhancer, forward 5′‐CATACAGAAATGAAGATGGG‐3′ and reverse 5′‐CATTTAATAAGTTAACAAGA‐3′;

for the 603 bp enhancer GATA binding site mutation, pair 1 for first site, forward 5′‐CTTCCAGTAGAAAAATATACGGTGGCCCACACCCCCAGTG‐3′ and reverse 5′‐GGGGTGTGGGCCACCGTATATTTTTCTACTGGAAGTATTTATGG‐3′; pair 2 for second site, forward 5′‐TTCACATACAAATAATATACGGTCAGGGTCAGGATCCAC‐3′ and reverse 5′‐ATCCTGACCCTGACCGTATATTATTTGTATGTGAATTTCC‐3′;

for the 603 bp enhancer GATA binding site mutation, forward 5′‐CGTTTGACCTAAAGATCCTTACAAGTATGAGTTTATCATATG‐3′ and reverse 5′‐AACTCATACTTGTAAGGATCTTTAGGTCAAACGATAATTTAG‐3′.

### 4.3 Cryosection and Eosin Staining

Embryos were fixed in 4% PFA in PBS at 4°C for 16 h following X‐gal staining. After fixation, embryos were washed three times in PBS (5 min each, room temperature) and sequentially cryoprotected in 10%, 20%, and 30% sucrose in PBS. Samples were then embedded in O.C.T. compound (Tissue‐Tek O.C.T. compound, SAKURA Finetechnical Co. Ltd., 4583) and frozen for cryosectioning.

Cryosections were cut at a thickness of 12 μm using a cryostat (Leica Biosystems), mounted on glass slides, and air‐dried at 37°C for 30 min, followed by three washes in PBS (10 min each) to remove residual O.C.T. Sections were dehydrated through graded ethanol (70%, 90%, and 99%; 3 s each) and stained with eosin solution (1:5 dilution of pure eosin; Muto Pure Chemicals, No. 32042) in ethanol for 10 min. After brief dehydration in 99% ethanol (3 s), sections were mounted with Fluoromount/Plus (Diagnostic Biosystems, K048). Images were acquired using a light microscope (Keyence).

## Author Contributions

**Yukihiro Harada:** methodology, data curation, investigation, validation, visualization, writing – review and editing, conceptualization. **Teruhisa Kawamura:** conceptualization, funding acquisition, writing – review and editing, project administration, supervision. **Tomoe Ueyama:** methodology, investigation, writing – review and editing. **Osamu Nakagawa:** conceptualization, funding acquisition, project administration, writing – review and editing. **Yunce Wang:** investigation, methodology, data curation, validation, visualization, writing – original draft, writing – review and editing. **Daiki Seya:** methodology, investigation.

## Funding

This work was supported in part by the grants from Japan Society for the Promotion of Science (Grants‐in‐Aid for Scientific Research, Grant Numbers JP21H02890 (to O.N., T.K., and Y.H.), JP23K18268 (to O.N., T.K., and Y.H.), JP24K02430 (to O.N., T.K., and Y.H.), and JP24K22041 (to T.K., Y.H. and T.U.)).

## Conflicts of Interest

The authors declare no conflicts of interest.

## Supporting information


**Figure S1:** No enhancer activity was detected by X‐gal staining at E7.5 in line 1.
**Figure S2:** X‐gal staining at E12.5 in the transgenic mouse line 2 showed enhancer activity restricted to the OFT, with no activity in the atrium.
**Figure S3:** The Enhancer candidates shorter than 603 bp did not exhibit reproducible reporter activity in F0 embryos.

## Data Availability

The data in this study are available from the corresponding authors upon reasonable request.
