# Supplementary material for: An Outflow Tract Myocardium‐Specific Enhancer at the Sema3c Locus During Heart Development
Source: Genes Cells. 2026 Jun 21;31(4):e70130. doi: 10.1111/gtc.70130 (PMC13283761; doi:10.1111/gtc.70130)
Supplement: Supplementary file 1 — Figure S1: No enhancer activity was detected by X‐gal staining at E7.5 in line 1. Figure S2: X‐gal staining at E12.5 in the transgenic mouse line 2 showed enhancer activity restricted to the OFT, with no activity in the atrium. Figure S3: The Enhancer candidates shorter than 603 bp did not exhibit reproducible reporter activity in F0 embryos. [file GTC-31-0-s001.pdf]

# Figure S1

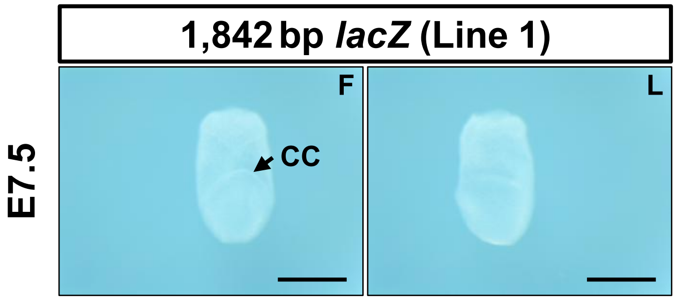


**No enhancer activity was detected by X-gal staining at E7.5 in line 1.**
X-gal staining was performed at 37 ℃ for 24 h.
cc, cardiac crescent; L, left view; F, front view. Scale bar: 500 μm

# Figure S2

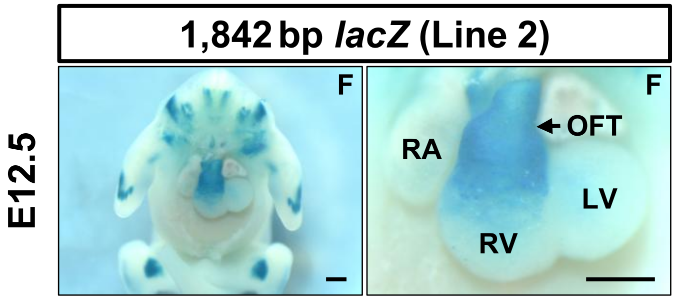


**X-gal staining at E12.5 in the transgenic mouse line 2 showed enhancer activity restricted to the OFT, with no activity in the atrium.**
X-gal staining was performed at 37 °C for 24 h.
OFT, outflow tract; A, atrium; LV, left ventricle; RV, right ventricle; F, front view.
Scale bar: 500 μm.

# Figure S3

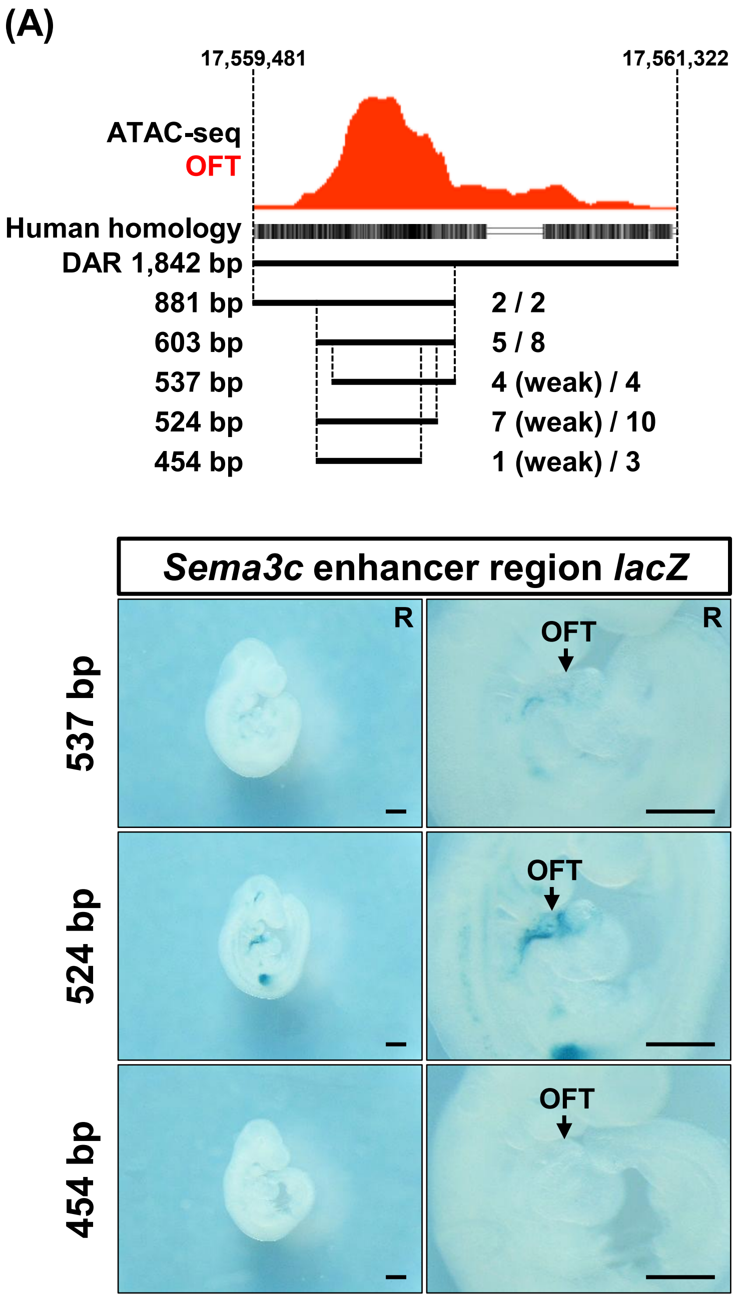


**The Enhancer candidates shorter than 603 bp did not exhibit reproducible reporter activity in F0 embryos.**

537 bp and 524 bp constructs showed only weak and discontinuous OFT staining in F0 embryos. The 454 bp construct showed no enhancer activity.

X-gal staining was performed at 37 °C for 24 h.

OFT, outflow tract; R, right view. Scale bar: 500 µm.
